# Integrated Metabolome and Transcriptome Analyses Reveal Etiolation-Induced Metabolic Changes Leading to High Amino Acid Contents in a Light-Sensitive Japanese Albino Tea Cultivar

**DOI:** 10.3389/fpls.2020.611140

**Published:** 2021-01-18

**Authors:** Hiroto Yamashita, Yuka Kambe, Megumi Ohshio, Aya Kunihiro, Yasuno Tanaka, Toshikazu Suzuki, Yoriyuki Nakamura, Akio Morita, Takashi Ikka

**Affiliations:** ^1^Faculty of Agriculture, Shizuoka University, Shizuoka, Japan; ^2^United Graduate School of Agricultural Science, Gifu University, Gifu, Japan; ^3^Tea Research Center, Shizuoka Prefectural Research Institute of Agriculture and Forestry, Shizuoka, Japan; ^4^Graduate Division of Nutritional and Environmental Science, Tea Science Center, University of Shizuoka, Shizuoka, Japan; ^5^Institute for Tea Science, Shizuoka University, Shizuoka, Japan

**Keywords:** albino, etiolation, tea plant, amino acids, transcriptome, metabolome, integrated omics

## Abstract

Plant albinism causes the etiolation of leaves because of factors such as deficiency of chloroplasts or chlorophylls. In general, albino tea leaves accumulate higher free amino acid (FAA) contents than do conventional green tea leaves. To explore the metabolic changes of etiolated leaves (EL) in the light-sensitive Japanese albino tea cultivar “Koganemidori,” we performed integrated metabolome and transcriptome analyses by comparing EL with green leaves induced by bud-sport mutation (BM) or shading treatments (S-EL). Comparative omics analyses indicated that etiolation-induced molecular responses were independent of the light environment and were largely influenced by the etiolation itself. Kyoto Encyclopedia of Genes and Genomes (KEGG) enrichment and pathway analyses revealed the downregulation of genes involved in chloroplast development and chlorophyll biosynthesis and upregulation of protein degradation-related pathways, such as the ubiquitin-proteasome system and autophagy in EL. Metabolome analysis showed that most quantified FAAs in EL were highly accumulated compared with those in BM and S-EL. Genes involved in the tricarboxylic acid (TCA) cycle, nitrogen assimilation, and the urea cycle, including the drastically downregulated *Arginase-1* homolog, which functions in nitrogen excretion for recycling, showed lower expression levels in EL. The high FAA contents in EL might result from the increased FAA pool and nitrogen source contributed by protein degradation, low N consumption, and stagnation of the urea cycle rather than through enhanced amino acid biosynthesis.

## Introduction

Albinism in plants is manifested as a phenotype of chlorotic or etiolated leaves (EL; i.e., white or yellow coloration, respectively) caused by deficiency of chloroplasts or chlorophylls. Many albino mutants have been identified in diverse plants, such as *Arabidopsis* (Motohashi et al., 2012), rice (Wu et al., 2011), tobacco (Bae et al., 2001), and tomato (Ishiga et al., 2009). Albinism phenotypes are caused by various factors, including environmental stress factors and abnormalities in genes involved in chloroplast development and/or chlorophyll biosynthesis (Kumari et al., 2009). In *Arabidopsis* albino or pale green leaf mutants, a difference in metabolic profiles associated with disruption of the internal membrane structure of chloroplasts is observed (Satou et al., 2014). However, less attention has been paid to metabolic dynamics in albino plants, although many studies of plant albinism have focused on direct factors causing albinism and the physiological functions involved in photosynthesis.

Tea plants (*Camellia sinensis* L.) are cultivated predominantly in Asia for production of green, oolong, and black tea, which are popular beverages throughout the world. Approximately 2 billion cups of tea are consumed daily worldwide (Drew, 2019), and drinking tea reportedly has numerous health benefits (Zhang et al., 2019). In general, tea quality is defined as the balance of the *umami* taste components such as free amino acids (FAAs) including theanine (Thea) and bitter taste components such as catechins and caffeine. In tea plantations, bud-mutants (bud-sports) with albino (chlorotic or etiolated) leaves are occasionally observed. Albino tea leaves generally contain higher FAA contents than those of conventional green tea leaves (Morita et al., 2011; Du et al., 2017; Cheng et al., 2018; Lu et al., 2018). Accumulation of high quantities of FAAs in the leaves improves the drinking quality of the brewed tea. Therefore, tea farmers have cloned albino mutants by cuttings to cultivate them as a unique cultivar and to take advantage of higher prices. Albino tea cultivars can be classified into two main types: temperature sensitive and light sensitive (Zhang et al., 2020a). The Chinese cultivars “Anjibaicha,” “Qiannianxue,” and “Xiaoxueya” are the most important temperature-sensitive cultivars in cultivation, and the causes of albinism in these cultivars have been well studied. These cultivars develop “snow-white” new leaves under a low temperature (<20°C) but greenish leaves under higher temperatures (22–25°C; Du et al., 2017). “Huangjinya,” “Baijiguan,” and “Yujinxiang” are the predominant cultivars of light-sensitive tea in cultivation. Their leaves change to green with a decrease in light intensity or under shading (Wu et al., 2016; Liu et al., 2017; Zhang et al., 2017), but these cultivars are poorly studied.

Omics approaches, such as metabolomics and transcriptomics, have become powerful tools for systematic monitoring of cellular responses (Fukushima and Kusano, 2014). In particular, integrated analysis of multiple omics data aids discovery and elucidation of underlying molecular mechanisms of complex traits. The major breakthrough in transcriptomics came with next-generation sequencing-based RNA sequencing (RNA-seq), which has allowed transcriptome analysis in species without prior availability of genomic information (Wang et al., 2009). Additionally, metabolomics has been extensively used as a principal tool for systems biology and molecular breeding as a result of technological advances, especially in improved mass resolution and sensitivity, high-throughput analysis, chromatography and analytical methodologies, and increasing availability of bioinformatics tools and databases (Hall et al., 2002; Bino et al., 2004; Baran et al., 2009; Quanbeck et al., 2012; Rhee, 2013; Rai et al., 2017; Nakabayashi and Saito, 2020). Metabolomics directly provides an understanding of the association between metabolism and certain biological events. Integration of metabolomics and transcriptomics has contributed to understanding the regulation of plant metabolism, such as the identification of specific responses and coordinate regulation in glucosinolate metabolism to sulfur or nitrogen deficiency in *Arabidopsis* (Hirai et al., 2004), the understanding of cultivar differences in response to cold stress in tobacco (Jin et al., 2017), and metabolic reprogramming under different light qualities and intensities in leaf lettuce (Kitazaki et al., 2018).

Recently, integrated metabolome and transcriptome analyses of the light- and temperature-sensitive Chinese albino tea cultivars “Huangjinya” (Zhang et al., 2017) and “Baiye 1” (Zhang et al., 2018), respectively, revealed a potential mechanism for coordinated regulation of carbon and nitrogen (N) metabolism. However, these previous studies (Zhang et al., 2017, 2018) are limited by the fact that each EL is compared to a different environmental condition of light and temperature or different genotype. Most of the omics-based previous studies on albino tea leaves have focused on some N metabolism, such as the tricarboxylic acid (TCA) cycle, and have not comprehensively analyzed key primary metabolisms, such as the urea cycle and photosynthesis-related and specialized metabolisms. In addition, the genes expressed in EL are not accurately listed because most transcriptome analyses are in *de novo*-based RNA-seq, not reference genome based. Therefore, the previous omics analyses have not explained the detailed mechanisms leading to metabolic changes in EL.

The present study was designed to examine the metabolic changes of EL in the light-sensitive Japanese albino tea cultivar “Koganemidori” by comparing them with green leaves induced by bud-sport mutation (BM) and/or shading treatment based on metabolomic and transcriptomic analyses. Our discovery of BM allowed for comparative analyses independent of differences in genotype and light environment in EL. The recent draft genome sequencing in tea plants (Wei et al., 2018) allowed us to perform the reference genome-based RNA-seq. The results indicated that the high FAA contents in EL were caused by promotion of the protein degradation pathway, low N consumption, and stagnation of the urea cycle rather than by enhanced amino acid biosynthesis.

## Materials and Methods

### Plant Materials and Chlorophyll Meter (SPAD) Measurement

Four Japanese albino tea cultivars, “Koganemidori,” “Hoshinomidori,” “Kiraka,” and “Yamabuki,” and a leading green tea cultivar in Japan, “Yabukita,” were investigated in this study. Mature tea ridges of “Koganemidori,” “Hoshinomidori,” and “Yabukita” were managed at the Tea Research Center, Shizuoka Prefectural Research Institute of Agriculture and Forestry, Kikugawa, Shizuoka, Japan. Mature tea ridges of “Kiraka” and “Yamabuki” were managed in private tea plantations at Fukuroi and Shizuoka, respectively, in Shizuoka Prefecture. New shoots of each albino tea cultivar in the four crop seasons, namely, spring (first), early summer (second), midsummer (third), and autumn (fourth), were plucked at three sites in tea ridges to measure the SPAD value. The average temperature in each sampling season in Shizuoka, Japan, was 14°C for spring, 23°C for early summer, 25°C for midsummer, and 19°C for autumn. The SPAD value of the third leaf from the shoot tip at the four-leaf stage of new shoots was measured using a SPAD-502Plus absorbance-based chlorophyll meter (Konica Minolta Inc., Osaka, Japan). The new leaves of “Yabukita” were also used as a reference.

Mature tea ridges of “Koganemidori” and its BM were shaded with an 85%-black-shade cloth (85P, Dio Chemicals, Tokyo, Japan) for 14 days in the first crop season in spring. The tea canopy was indirectly covered by the shade cloth. After shade treatment, at the development of the fourth leaf of new shoots, the shoots were plucked from approximately eight sites in one replicate from nonshaded and shaded tea ridges. The plucked shoots with three replicates were immediately separated into individual leaves, frozen in liquid N, and stored at −80°C until subsequent analyses. Some of the frozen samples were freeze-dried, ground to a fine powder, and stored at room temperature in the dark for subsequent chemical component analysis.

The experiment was conducted in 2015 and 2017, but the shading treatment of BM was not conducted in 2015. Each sample was defined as EL, BM, shaded EL (S-EL), and shaded BM (S-BM). In 2015, the dried samples were used for analysis of FAAs, catechins, and caffeine contents. In subsequent catechins and caffeine measurements, for the fourth new leaves of S-EL, only two replicates could be performed because of the small sample volume. Before plucking, the bottom leaf of new shoots at the four-leaf stage was used to measure photosynthesis activity. In 2017, the frozen samples were used for metabolome and transcriptome analyses, and the dried samples were used to analyze the chlorophyll content.

### Measurement of Chlorophyll Content

Chlorophylls *a* and *b* were extracted from finely ground powder (5 mg) of freeze-dried leaf samples using *N*,*N*'-dimethylformamide (5 ml). After incubation for 24 h at 4°C in the dark to allow complete decolorization, the samples were centrifuged at 2,000 × *g* for 30 min, and the absorbance of the supernatant was measured at 663.8 and 646.8 nm using a spectrophotometer (UV-1800, Shimadzu, Kyoto, Japan). Chlorophyll *a* and *b* contents were calculated using the equation of Porra et al. (1989), and their total value was expressed as a phenotype of chlorophyll content.

### Measurement of Photosynthetic Activity

The photosynthetic rate was measured with a portable photosynthesis system (LI-6400XT, LI-COR, Lincoln, NE, United States). The conditions in the leaf chamber were set as follows: photosynthetic photon flux density 1,500 *μ*mol m^−2^ s^−1^, CO_2_ concentration 380 μmol mol^−1^, and block temperature 25°C. Photosynthesis was measured from 10:00 to 14:00. The net photosynthetic rate was expressed as the rate of CO_2_ uptake. Each leaf was measured three times to account for measurement errors, and the average value was taken as the measured value for each leaf.

### Quantification of FAA Content

Dry ground plant tissue (10 mg) was added to 10 mg of polyvinylpolypyrrolidone and 5 ml of ultrapure water and extracted by shaking (130 strokes per minute) for 60 min at room temperature. After centrifugation at 2,000 × *g* for 15 min at 4°C, the supernatant was passed through a 0.45-μm cellulose acetate filter (ADVANTEC, Tokyo, Japan). The filtered solution was stored at −30°C until high-performance liquid chromatography (HPLC) analysis. Homoserine, as an internal standard, was added to the filtered solution, and *o*-phthalaldehyde derivatives were analyzed using HPLC. The HPLC system (Shimadzu, Tokyo, Japan) comprised two LC-10AT pumps, a DGU-20A5R degasser, a CTO-10Avp column oven, a Prominence RF-20A fluorescence detector, an SCL-10Avp system controller, and an SIL-10AF autosampler. The HPLC conditions were as follows: injection volume, 5 μl; column, 150 mm × 4.6 mm × 5 μm Develosil ODS-HG-5 (Nomura Chemical Co., Ltd., Aichi, Japan); temperature of the column oven, 40°C; excitation wavelength, 340 nm; and emission wavelength, 450 nm. Eluent A [5 mM citrate buffer, pH 6.0, and 5% (v/v) acetonitrile] and eluent B [5 mM citrate buffer, pH 6.0, and 70% (v/v) acetonitrile] were used as the mobile phases at a flow rate of 1.0 ml min^−1^. The elution was performed with the following gradient: initial concentration of 5.0% B, followed by 0.1 min hold at 5.0%, 4.9 min linear gradient from 5.0 to 12% B, 15 min linear gradient from 12 to 22% B, 5.0 min linear gradient from 22 to 95% B, 5.0 min hold at 95% B, 1.0 min linear gradient from 95 to 5.0% B, and 5.0 min linear gradient from 5.0 to 0%. The solution of this mobile phase was eluted for 36 min per sample. Eight major FAAs in tea leaves (Mukai et al., 1992; Asp, aspartic acid; Asn, asparagine; Glu, glutamic acid; Gln, glutamine; Arg, arginine; Thea, theanine; Ala, alanine; GABA, gamma-aminobutyric acid) were used as standards and quantified.

### Quantification of Catechins and Caffeine Contents

Dry ground plant tissue (25 mg) was added to 5 ml of 50% acetonitrile and extracted by shaking (130 strokes per minute) for 60 min at room temperature. After centrifugation at 2,000 × *g* for 15 min at 4°C, the supernatant was passed through a 0.45-μm polytetrafluoroethylene filter (ADVANTEC, Tokyo, Japan). The filtered solution was stored at −30°C until HPLC analysis. The HPLC system (Shimadzu, Tokyo, Japan) comprised two LC-10ADvp pumps, a DGU-14A degasser, a CTO-20 AC column oven, a Prominence SPD-M20A photodiode array detector, an SCL-10Avp system controller, and an SIL-10ADvp autosampler. The HPLC conditions were as follows: injection volume, 5 μl; column, 150 mm × 4.6 mm × 5 μm Develosil RPAQUEOUS-AR-5 (Nomura Chemical Co., Ltd., Aichi, Japan); temperature of the column oven, 40°C; and photodiode array detector, 190–400 nm. Eluent A [400:10:1 ml (v/v/v), ultrapure water: acetonitrile: 85% phosphoric acid] and eluent B [2:1 ml (v/v/v), Eluent A: methanol] were used as the mobile phases at a flow rate of 1.0 ml min^−1^. The elution was performed with the following gradient: initial concentration of 1.0% B, followed by 2.0 min hold at 1.0% B, 43 min linear gradient from 1.0 to 80% B, 5.0 min hold at 80% B, 10 min linear gradient from 80 to 1.0% B, and a final concentration of 1.0% B for 5.0 min. The solution of this mobile phase was eluted for 65 min per sample. Six catechins [EC, (−)-epicatechin; ECG, (−)-epicatechin gallate; EGC, (−)-epigallocatechin; EGCG, (−)-epigallocatechin gallate; GC, (+)-gallocatechin; and (+)C, (+)-catechin] and caffeine were used as standards and quantified.

### Metabolome Analysis Based on Capillary Electrophoresis-Time-of-Flight Mass Spectrometry

The metabolome analysis was conducted by Human Metabolome Technologies, Inc (Yamagata, Japan). Frozen ground samples (30 mg) were transferred to a tube containing 600 μl of methanol and 50 μM of an internal standard from Human Metabolome Technologies, Inc. After homogenization, 600 μl of chloroform and 240 μl of ultrapure water were added to the homogenate, mixed well, and centrifuged at 2,300 × *g* for 5 min at 4°C. The resultant water phases were subjected to ultrafiltration using a Millipore Ultrafree-MC centrifugal filter device 5 kDa (Millipore, Billerica, MA, United States) and then centrifuged at 9,100 × *g* for 120 min at 4°C. The filtrates were dried, dissolved in 50 μl of ultrapure water, and subjected to CE-TOFMS analysis using an Agilent CE-TOFMS system (Agilent, Palo Alto, CA, United States) following a previously described method (Soga and Neiger, 2000; Soga et al., 2002, 2003). The 171 metabolites detected in at least one sample were used for subsequent analyses. Principal component analysis (PCA) was performed based on normalized data for the relative peak area to confirm reproducibility among replicates using the “prcomp” function in R software ver. 4.0.2 (R Core Team, 2020). Differentially accumulated metabolites (DAMs) were detected based on multiple testing with the Benjamini-Hochberg false discovery rate (FDR) from *p*-values obtained with Welch’s *t*-test using the “*t*.test” and “*p*.adjust” functions in R software ver. 4.0.2 (R Core Team, 2020). A cutoff of FDR < 0.05 was applied as DAMs in the subsequent analysis.

### Transcriptome Analysis Based on RNA-Seq

Total RNA was extracted using the RNeasy Plant Mini Kit (Qiagen, Valencia, CA, United States) in accordance with the manufacturer’s instructions. During RNA extraction, DNase treatment was not used because it did not affect the library preparation method. The RNA concentration was measured using a Qubit fluorometer (Thermo Fisher Scientific, Waltham, MA, United States), and the RNA quality was assessed using a Bioanalyzer (Agilent). RNA samples with RNA integrity >8.0 were used for subsequent RNA-seq. Library preparation was performed using a KAPA Stranded mRNA-Seq Kit (Kapa Biosystems, Wilmington, MA, United States) in accordance with the manufacturer’s instructions. Sequencing was performed using a HiSeq 4,000 platform with the TruSeq SBS Kit v3-HS (Illumina, San Diego, CA, United States) in paired-end mode with a read length of 100 bp. Each experiment was performed with three biological replicates for each sample. The sequence data in this RNA-seq have been deposited in the DNA Data Bank of Japan (DDBJ) Sequence Read Archive (accession number: DRA010755).

Reads were preprocessed using Trimmomatic-0.39 with the following parameters: ILLUMINACLIP TruSeq3-PE-2.fa:2:30:10, LEADING:15, TRAILING:15, SLIDINGWINDOW:10:15, and MINLEN:50. After preprocessing, the remaining reads were mapped to the tea reference draft genome (Wei et al., 2018), which was downloaded from the Tea Plant Information Archive (TPIA; Xia et al., 2019), using STAR ver. 2.7.3a (Dobin et al., 2013). Gene expression levels were estimated using RSEM ver. 1.3.3 (Li and Dewey, 2011). The output of RSEM was subsequently analyzed using R software ver. 4.0.2 (R Core Team, 2020). Genes with zero counts in at least one sample were excluded, and 20,137 genes were used for subsequent analyses. PCA was performed based on transcripts per million to confirm the reproducibility among replicates using the “prcomp” function in R software ver. 4.0.2 (R Core Team, 2020).

Differentially expressed genes (DEGs) were detected using the R package “edgeR” ver. 3.30.3 (Robinson et al., 2010) as follows. The count data of the remaining genes were normalized using the trimmed mean of the M-values method with the function “calcNormFactors.” The function “exactTest” was performed to calculate the log-transformed fold change (log_2_FC) and the FDR. The cutoff of FDR < 0.05 was applied as DEGs in the subsequent analysis. Kyoto Encyclopedia of Genes and Genomes (KEGG) pathways and gene ontology (GO) enrichment analyses of the DEGs were performed using TeaCoN, a gene co-expression network database for tea plants (Zhang et al., 2020b) with a cutoff FDR of <0.05. Pathway analysis of DAMs and DEGs was constructed based on the KEGG pathway. The genes used for the pathway analyses are summarized in Supplementary Table S1. Public tissue transcriptome data (Wei et al., 2018) were downloaded from TPIA (Xia et al., 2019).

## Results

### Seasonal or Shading-Induced Changes of Leaf Color in Four Japanese Albino Tea Cultivars

We investigated the seasonal changes of leaf color in four albino tea cultivars in Japan from spring (the first crop season) to autumn (the fourth crop season). The EL of the temperature-sensitive cultivars “Kiraka,” “Hoshinomidori,” and “Yamabuki” were green from early summer (the second crop season) to autumn (the fourth crop season; Supplementary Figure S1). Conversely, the EL of the light-sensitive cultivar “Koganemidori” were maintained from spring (the first crop season) to autumn (the fourth crop season; Supplementary Figure S1).

**Figure 1:**
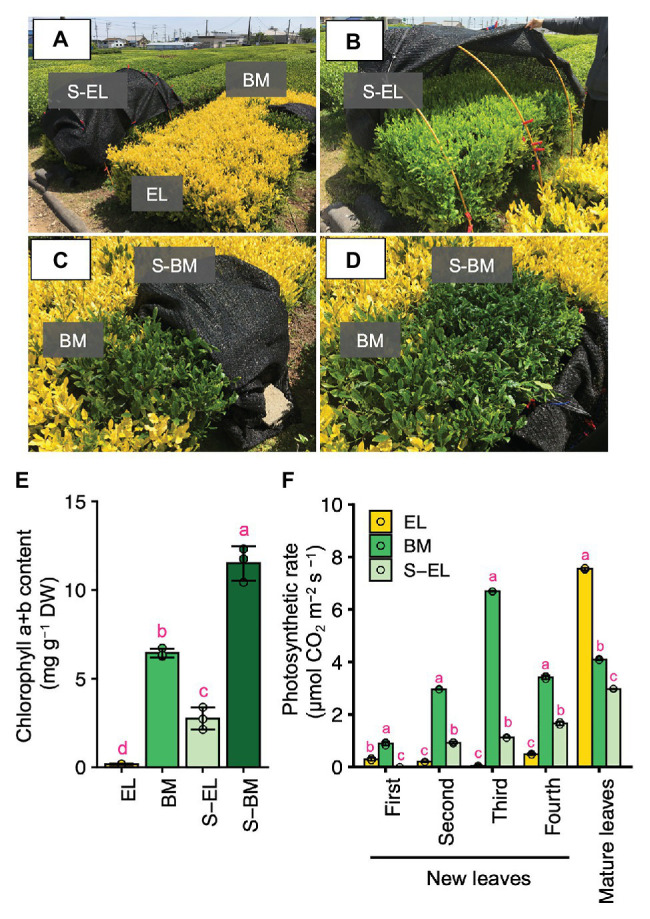
Green phenotype induction in etiolated leaves (EL) of “Koganemidori.” Phenotype of the albino tea cultivar “Koganemidori” **(A)**. Phenotype of EL after shading treatment **(B)**. Green leaves resulting from a bud-sport mutation (BM) in the tea ridge of “Koganemidori” **(C)**. Phenotype of BM after shading treatment (S-BM; **D**). Chlorophyll content in the third new leaf of etiolated leaves (EL), BM, shaded EL (S-EL), and S-BM at the first crop in 2017 **(E)**. Photosynthetic rate of four leaves by leaf position in new shoots and mature leaves of EL, BM, S-EL, and S-BM at the first crop in 2015 **(F)**. Data and error bars are the mean ± SD (*n* = 3). Statistical tests were performed based on Tukey’s honestly significant difference (HSD) test using the “glht” function of the R package “multcomp” ver. 1.4–13. Different letters above bars indicate a significant difference in each part of new shoots (Tukey’s HSD test, *p* < 0.05).

On albino tea plants, only the newly emergent leaves are etiolated, whereas the mature leaves are green. Thus, the mature leaf color of “Koganemidori” was green (visual observation; Figure 1A). In a tea ridge of new EL of “Koganemidori,” we observed a BM in which the leaves reverted to green as a natural mutation (Figure 1A). The EL changed to green under shading treatment (Figures 1A,B,E). Additionally, the chlorophyll content in green leaves of BM was increased in response to shading treatment (Figures 1C–E). The photosynthetic rate of EL was low, whereas that of mature leaves was high, compared with the photosynthetic rates of BM and S-EL (Figure 1F).

### Characterization of Tea Quality-Related Metabolites in EL

**Figure 2:**
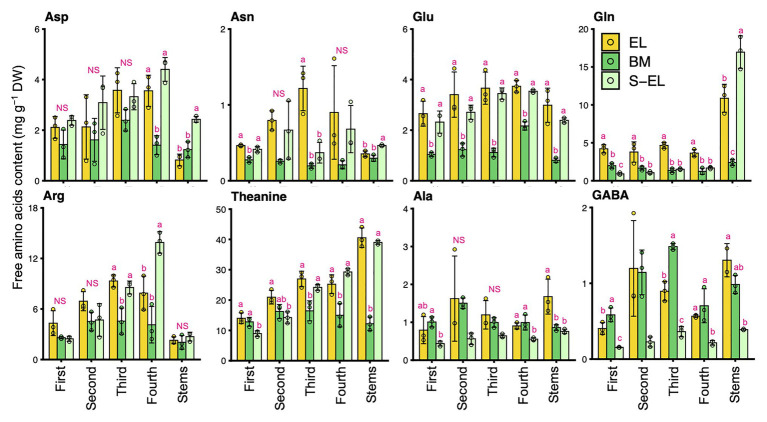
Effect of green phenotype induction on free amino acid content in new leaves and stems of the light-sensitive albino tea cultivar “Koganemidori.” The free amino acid contents in leaves at each leaf position (first, second, third, and fourth) and stems of new shoots at the first crop season in 2015 were analyzed by high-performance liquid chromatography (HPLC). Data and error bars are the mean ± SD (*n* = 3). Statistical tests were performed based on Tukey’s honestly significant difference (HSD) test using the “glht” function of the R package “multcomp” ver. 1.4–13. Different letters above bars indicate a significant difference in each part of new shoots (Tukey’s HSD test, *p* < 0.05). NS: not significant (*p* ≥ 0.05).

**Figure 3:**
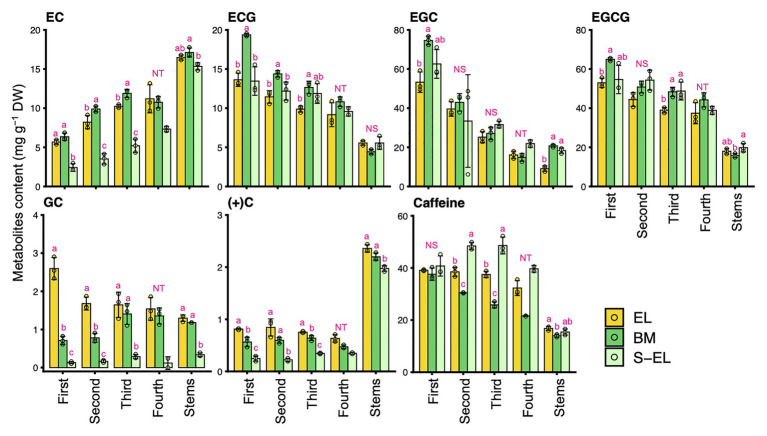
Effect of green phenotype induction on catechin and caffeine contents in new leaves and stems of the light-sensitive albino tea cultivar “Koganemidori.” The catechin and caffeine contents in leaves at each leaf position (first, second, third, and fourth) and stems of new shoots at the first crop season in 2015 were analyzed by high-performance liquid chromatography (HPLC). Data and error bars are the mean ± SD (*n* = 3). The fourth new leaves in shaded EL (S-EL) comprised only two replicates. Statistical tests were performed based on Tukey’s honestly significant difference (HSD) test using the “glht” function of the R package “multcomp” ver. 1.4–13. Different letters above bars indicate a significant difference in each part of new shoots (Tukey’s HSD test, *p* < 0.05). NS: not significant (*p* ≥ 0.05). NT: not tested.

To characterize the chemical components of the EL of “Koganemidori,” we analyzed the contents of eight FAAs (Asp, Asn, Glu, Gln, Arg, Thea, Ala, and GABA), six catechins [EC, ECG, EGC, EGCG, GC, and (+)C], and caffeine as major tea quality-related metabolites by HPLC. Figure 2 shows the FAA contents in new leaves and stems of EL, BM, and S-EL at the first crop in 2015. In most cases, the FAA contents in EL were significantly higher, especially in the third and fourth mature leaves, than those in BM. In new stems, the Glu, Gln, Thea, and Ala contents in EL were significantly higher than those in BM. The catechins and caffeine contents in EL, BM, and S-EL in the first crop are shown in Figure 3. In most leaves, especially the first and third leaves, the EC, ECG, EGC, and EGCG contents in EL were significantly lower than those in BM. The EC and GC contents in EL were decreased by shading. In new stems, the EGC content in EL was lower than that in BM. The caffeine content in EL was higher than that in BM and was increased in the second and third leaves by shading.

### Integrated Omics Analyses Based on Metabolome and Transcriptome Data

**Figure 4:**
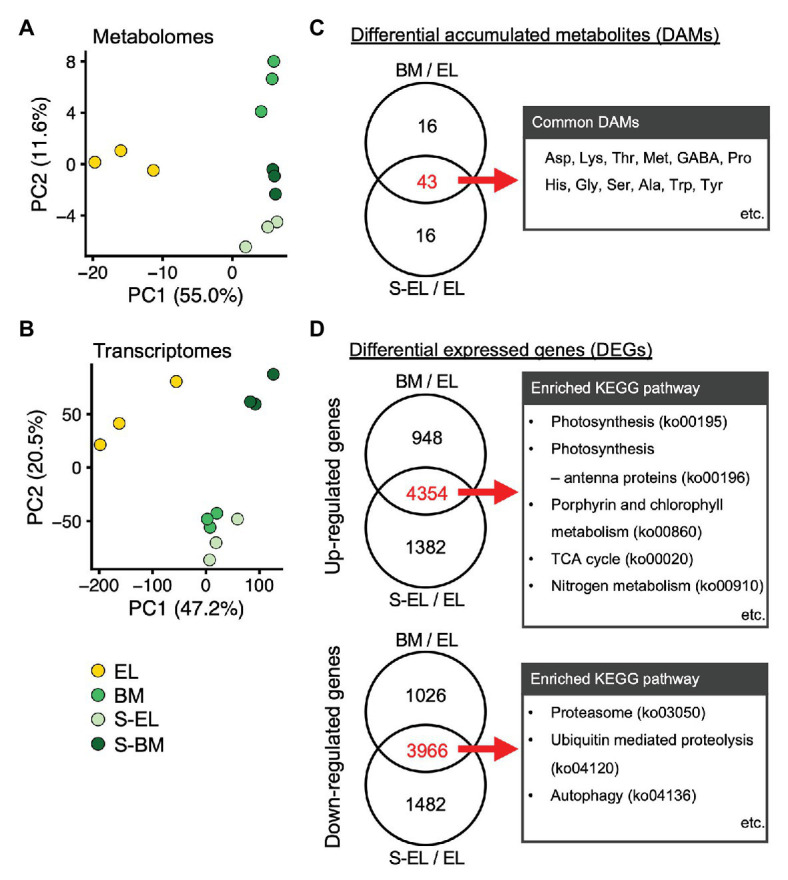
Profiles of metabolomes and transcriptomes in etiolated tea leaves (EL) and under green phenotype induction by bud-sport mutation (BM) and shading treatment (S-EL). Principal component analysis (PCA) of the metabolome **(A)** and transcriptome **(B)** of EL under green phenotype induction. PC1 and PC2 scores were plotted to assess the reproducibility among the three replicates for all samples. Venn diagram analyses of differentially accumulated metabolites (DAMs; **C**) and differentially expressed genes (DEGs; **D**) in comparisons between BM/EL and S-EL/EL. Representative metabolites and enriched Kyoto Encyclopedia of Genes and Genomes (KEGG) pathways in DAMs and DEGs in common between BM/EL and S-EL/EL are listed to the right of the Venn diagrams.

To understand the molecular mechanism of etiolation and the induced metabolic changes in a light-sensitive albino tea cultivar, we conducted analyses of the metabolome and transcriptome of each “Koganemidori” leaf in 2017. Metabolome analysis based on CE-TOFMS detected a total of 171 metabolites (84 in the cation mode and 87 in the anion mode; Supplementary Table S2). RNA-seq using a HiSeq 4,000 platform (with three replicates in each condition) yielded approximately 19–30 million raw reads per replicate (Supplementary Table S3). Of the preprocessed reads, approximately 97% passed quality control and were mapped to the reference genome with an approximately 98% mapping rate. Expression of 20,137 genes was detected in all replicates. PCA was performed to assess the reproducibility of the metabolomes and transcriptomes. Under the same conditions, data for all three replicates corresponded closely, and EL, BM, S-BM, and S-EL were separated on the first principal component in both omics analyses (Figures 4A,B). DAMs and DEGs were detected statistically on the basis of the FDR cutoff of <0.05 in the three comparisons, namely, EL vs. BM (BM/EL), EL vs. S-EL (S-EL/EL), and BM vs. S-BM (S-BM/BM), to reveal the effect of greening and shading. A Venn diagram revealed 43 DAMs in common between BM/EL and S-EL/EL (Figure 4C), but only two DAMs in common between S-EL/EL and S-BM/BM (Supplementary Figure S2). The common DAMs detected between BM/EL and S-EL/EL included numerous FAAs (Figure 4C; Supplementary Table S2). For upregulated and downregulated DEGs, Venn diagrams revealed the same pattern as described for DAMs (Figure 4D; Supplementary Figure S2). Upregulated DEGs in common between BM/EL and S-EL/EL were enriched in KEGG pathways such as “photosynthesis (ko00195)” and “porphyrin and chlorophyll metabolism (ko00860)” involved in photosynthesis and chlorophyll metabolism and “TCA cycle (ko00020)” and “nitrogen metabolism (ko00910)” involved in amino acid metabolism (Figure 4D). Downregulated DEGs in common between BM/EL and S-EL/EL were enriched in KEGG pathways such as “proteasome (ko03050),” “ubiquitin mediated proteolysis (ko04120),” and “autophagy (ko04136)” involved in protein degradation (Figure 4D). The GO terms that were enriched in upregulated or downregulated DEGs in common between BM/EL and S-EL/EL showed similar results to the KEGG pathway analysis (Supplementary Tables S4 and S5).

**Figure 5:**
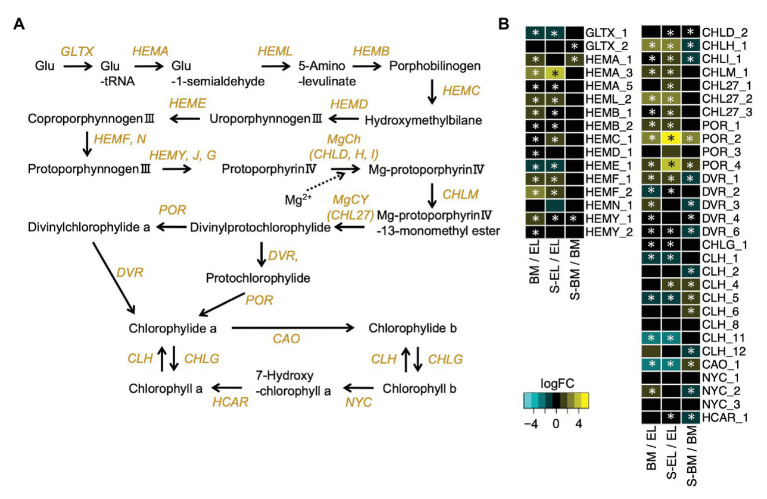
Expression profiles of genes involved in chlorophyll biosynthesis. Schematic presentation of the chlorophyll biosynthesis pathway **(A)** and expression profiles of genes involved in the pathway **(B)**. Heatmaps show log_2_ fold change (log_2_FC) of the expressed genes in the comparisons between BM/EL (left), S-EL/EL (middle), and S-BM/BM (right). An asterisk in the boxes indicates a significant difference [false discovery rate (FDR) < 0.05].

We conducted pathway analysis of DEGs involved in chloroplast development and chlorophyll metabolism. The *Golden2-like* (*GLK*) genes encode transcription factors that regulate chloroplast development in diverse species (Fitter et al., 2002). The two homologs (*GLK1-1*, TEA009544; and *GLK1-2*, TEA015144) of the *GLK1* ortholog were significantly downregulated in EL compared with that in BM and S-EL, but the *GLK2* ortholog was upregulated in EL (Supplementary Figure S3). Genes that function in photosynthesis and light harvesting were also significantly downregulated in EL (Supplementary Figure S3). Additionally, most genes involved in chlorophyll biosynthesis, including the crucial genes *protochlorophyllide reductase* (*POR*) and *chlorophyll a synthase* (*CHLG*), were significantly downregulated in EL compared with that in BM and S-EL (Figures 5A,B). Most homologs of *chlorophyllase* (*CLH*), catalyzing the hydrolysis of chlorophyll, and *chlorophyllide a oxygenase* (*CAO*) were significantly upregulated in EL (Figures 5A,B).

**Figure 6:**
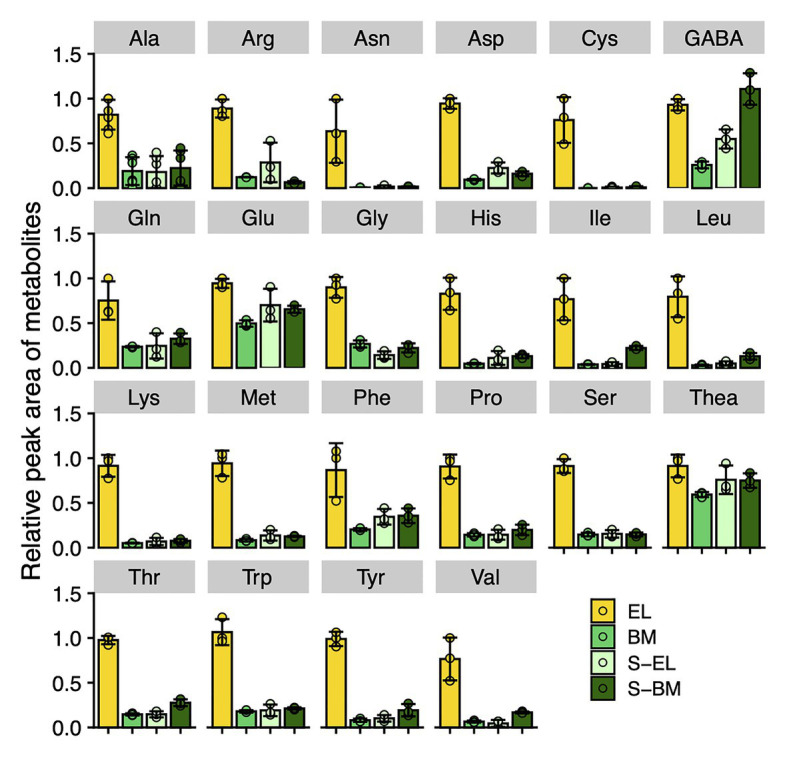
Metabolic changes of free amino acids in etiolated tea leaves (EL) and under green phenotype induction by bud-sport mutation (BM) and shading treatment (S-EL). The free amino acid contents in the third new leaf at the first crop season in 2017 were analyzed by capillary electrophoresis-time-of-flight mass spectrometry (CE-TOFMS). Ala means β-alanine. Data and error bars are the mean ± SD (*n* = 3).

**Figure 7:**
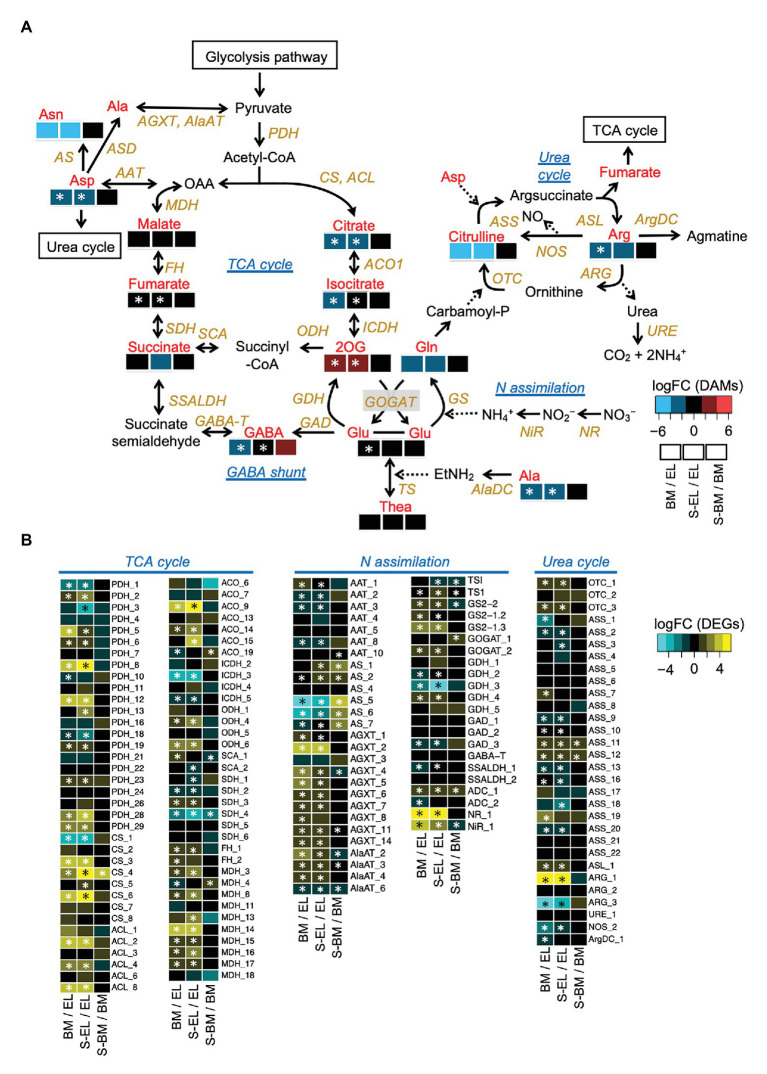
Etiolation-induced metabolic dynamics in the tricarboxylic acid (TCA) cycle, nitrogen assimilation, and the urea cycle based on an integrated pathway analysis of the metabolome and transcriptome in etiolated tea leaves (EL) and under green phenotype induction by bud-sport mutation (BM) and shading treatment (S-EL). The metabolism pathway was displayed with log_2_ fold change (log_2_FC) of metabolome data **(A)**. Expression profiles of genes involved in the pathway **(B)**. Heatmaps show log_2_ fold change (log_2_FC) of the metabolites and the expressed genes in the comparisons BM/EL (left), S-EL/EL (middle), and S-BM/BM (right). An asterisk in the boxes indicates a significant difference (FDR < 0.05).

Metabolome analysis based on CE-TOFMS indicated that most FAAs were highly accumulated in EL in contrast to BM, S-BM, and S-EL (Figure 6). We used an integrated omics approach to understand the metabolic changes of pathways leading to high FAA contents in EL. Many genes involved in the TCA cycle and N assimilation showed low expression levels in EL (Figure 7). With a focus on the urea cycle of the N excretion pathway for recycling, the downregulation of one *Arginase* (*ARG*) homolog (*ARG-1*, TEA009085), which was the most highly expressed of the three *ARG* homologs, was observed in EL (Figure 7; Supplementary Figure S4).

## Discussion

Albino tea leaves generally accumulate higher FAA contents than those of conventional green tea leaves (Morita et al., 2011; Du et al., 2017; Cheng et al., 2018; Lu et al., 2018). In the present study, we conducted an integrated metabolomic and transcriptomic approach to examine the metabolic changes of etiolation by comparing EL with green leaves (BM and S-EL) in a light-sensitive albino tea cultivar. Analyses of DAMs and DEGs showed that many molecules were shared among the greenish phenotypes (Figure 4). Although EL have hypersensitivity to light, our transcriptome data indicated that the common DEGs between BM/EL and S-EL/EL did not enrich the GO term associated with high light stress response (Mullineaux et al., 2018), such as response to reactive oxygen (GO:0000302), response to oxidative stress (GO:0006979), and so on (Supplementary Tables S4 and S5). These results indicated that the etiolation-induced molecular responses were independent of the light environment and were largely influenced by the etiolation itself. Analyses of KEGG pathways and GO enrichment among the common DEGs revealed that the expression of genes functioning in chloroplast development and chlorophyll biosynthesis was drastically lowered in EL compared with that in BM and S-EL (Figure 4; Supplementary Figure S3; Supplementary Tables S4 and S5). The *GLK* transcription factors regulate chloroplast development (Fitter et al., 2002). The *Arabidopsis glk1 glk2* double mutant shows a pale green leaf phenotype, and mesophyll cells contain small chloroplasts with sparse thylakoid membranes that fail to form grana (Yasumura et al., 2005; Waters et al., 2008). In EL of the albino tea cultivar “Koganemidori,” the expression of two *GLK1* orthologs (*GLK1-1* and *GLK1-2*) was drastically reduced, but that of the *GLK2* ortholog was not (Supplementary Figure S3). In the eudicot *Arabidopsis*, two *GLK* homologs, *GLK1* and *GLK2*, act redundantly to regulate monomorphic chloroplast development (Fitter et al., 2002). Conversely, in the monocot maize, each gene acts alone in one of the two photosynthetic cell types that develop in the leaf (Rossini et al., 2001). These results suggested that the *GLK1* homologs predominantly regulated chloroplast development in etiolation of albino tea leaves. Many genes involved in chlorophyll biosynthesis, including *POR* and *CHLG*, were downregulated in EL (Figures 5A,B). POR catalyzes the conversion of protochlorophyllide to chlorophyllide (Figure 5A) and is the only reaction that requires light in the chlorophyll pathway (Griffiths, 1978). The causal gene of the rice *faded green leaf* (*fgl*) mutant, which shows a yellow-green leaf phenotype, is a *POR* homolog (Sakuraba et al., 2013). Previous studies on an albino tea cultivar have observed reduced expression of *POR* and its encoding genes (Dong et al., 2018; Lu et al., 2018). These results suggest that etiolation of a light-sensitive albino tea cultivar might be caused by a redundant reduction of genes involved in chloroplast development and chlorophyll biosynthesis. A future challenge is to understand the regulatory mechanism of these genes, such as epigenetic regulation. For example, in a yellow fruit somatic mutant of apple (*Malus* × *domestica*), El-Sharkawy et al. (2015) observed that differences in DNA methylation in the promoter region might be an epigenetic factor responsible for the difference in fruit color.

The present HPLC and CE-TOFMS analyses showed that FAAs were more strongly accumulated in EL than they were in BM and S-EL (Figures 2, 6). Due to the annual (environmental) effect in the field and the differences of analytical methods, the trend in the FAA accumulation pattern in S-EL was slightly different between 2015 and 2017, but that pattern in BM was robust (Figures 2, 6). This result also indicated that FAAs were highly accumulated in EL under etiolation alone regardless of the light environment and that comparison of BM and S-EL green leaves, which share the same genetic background and environment as EL, is suitable as a comparative control to reveal metabolic changes in EL. This is an important point for understanding the metabolic changes because most previous studies (Morita et al., 2011; Du et al., 2017; Zhang et al., 2018) on albino tea leaves have been based on comparisons between an albino cultivar and a conventional green tea cultivar that differ in genetic backgrounds, which raises concerns about bias owing to the genetic differences. A comparative analysis of EL and shading-induced green leaves has also been performed, but it has not been possible to isolate physiological responses due to differences in light conditions (Zhang et al., 2017). The experimental design in this study with BM and shading treatment allowed us to eliminate the effects of the light conditions and genetic background and to evaluate the effects of metabolic changes in etiolation itself. We conducted an integrated pathway analysis on primary metabolism, namely, the TCA cycle, N assimilation, and the urea cycle, involved in amino acid biosynthesis. Many genes involved in the TCA cycle and N assimilation showed low expression levels in EL (Figure 7). Downregulation of *nitrate reductase* (*NR*) and *nitrite reductase* (*NiR*) involved in N assimilation and of genes in the glutamine synthetase/glutamate synthase (GS/GOGAT) cycle was observed in EL (Figure 7). *Theanine synthetase* (*TS*) genes were not upregulated in EL (Figure 7). The metabolites from glycolysis in EL accumulated at the same level as in BM and S-EL (Supplementary Figure S5). These phenomena in EL, which have been observed in a previous study (Zhang et al., 2017), might by caused by feedback inhibition in the TCA cycle and N assimilation metabolism due to the accumulation organic acids and amino acids. In addition, these results might indicate that the strong accumulation of FAAs in EL was not caused by the promotion of N assimilation and amino acid biosynthesis. In *Arabidopsis* albino or pale green leaf mutants, enhanced accumulation of several FAAs (especially Glu, Gln, and Asn) resulting from upregulation of N assimilation-related genes was observed (Satou et al., 2014). It was suggested that the albinism-induced FAA accumulation in tea plants and *Arabidopsis* might be caused by different mechanisms. In addition to the regulatory mechanisms of each metabolic pathway, one reason that an albino plant differs between tea and *Arabidopsis* plants is chimeras within the tree (the new leaves are albino and the mature leaves are green in a typical albino tea cultivar), which differ depending on the N sources for plant growth. However, in the present study, we detected the downregulation of *ARG-1*, which is the most highly expressed of the three *ARG* homologs in tea plants (Figure 7; Supplementary Figure S4). Although the upregulation of *ARG-3* was observed in EL, it was the lowest expressed of the three *ARG* homologs (Supplementary Figure S4). *ARG-1* was more highly expressed in new leaves than in mature and old leaves based on publicly available tissue transcriptome data for tea plants (Supplementary Figure S4; Wei et al., 2018). Arginase hydrolyzes arginine to urea and ornithine. An important role of mammalian ARG is elimination of waste N *via* the urea cycle. In contrast to this detoxification function, the coordinated activity of ARG and urease in plants provides a mechanism to recycle urea-N (Zonia et al., 1995; Todd and Gifford, 2002). These results suggest that the reduced N efflux for N recycling in the urea cycle by downregulation of *ARG* might diminish the metabolic flow of the urea cycle, which in turn may cause upstream accumulation of amino acids. Functional analysis of *ARG-1* in tea plants warrants further study.

The present KEGG pathways and GO enrichment analysis revealed the upregulation of protein degradation-related pathways, such as the ubiquitin-proteasome system (UPS) and autophagy in EL (Figures 4C,D; Supplementary Tables S4 and S5). The UPS and autophagy are evolutionarily conserved systems for degradation of proteins and organelles (Dikic, 2017). Chloroplasts are also a target of the UPS and autophagy (Ling et al., 2012; Ishida and Makino, 2018). The turnover of damaged chloroplasts is critical for management of oxidative damage and recycling of assimilated nutrients (Ishida and Makino, 2018). Two types of autophagy degrade chloroplasts: the Rubisco-containing body (RCB) pathway (Ishida et al., 2008; Izumi et al., 2015), by which autophagosomes transport a portion of the chloroplast stroma in a specific form enclosed in small bodies containing stromal proteins, and chlorophagy (Izumi et al., 2017), which involves vacuolar transport of entire chloroplasts. In particular, RCB-mediated autophagy is important in recycling amino acids during developmental growth or adaptation to sugar starvation (Izumi et al., 2013; Ono et al., 2013; Hirota et al., 2018). It is possible that chloroplast development in EL is abnormal, and therefore, autophagy of incomplete chloroplasts may be active. The N cost of Rubisco and light-harvesting complex chlorophyll-binding proteins of photosystems I and II (LHCs: LHCA and LHCB) is high in leaves (Evans and Seemann, 1989; Makino et al., 2003). These proteins are imported into chloroplasts. The LHCs bind pigments in chloroplasts, which are then protected against protein degradation (White and Green, 1987). However, the chlorophyll content was low in EL (Figure 1E); therefore, almost all LHCs could be degraded because they could not bind to chlorophyll. The degradation of LHCs might have contributed to the high FAA content in EL.

The predominant N-containing compounds in tea plants are protein, chlorophyll, FAAs, and caffeine. In the present quantitative analysis, the caffeine content in EL was higher than that in BM and S-EL or did not change (Figure 3; Supplementary Figure S6), although the many genes involved in caffeine biosynthesis were downregulated in EL (Supplementary Figure S6). These results suggest that, although low N consumption in chlorophyll biosynthesis might have contributed to the elevated accumulation of FAAs in EL, N consumption in caffeine biosynthesis was not a factor. We performed transcriptome analysis of the flavonoid and catechin biosynthesis pathway, a specialized secondary metabolism pathway in tea plants. Most genes involved in the general flavonoid pathway and those involved in synthesizing catechins, including *phenylalanine ammonia-lyase* (*PAL*), *chalcone synthase* (*CHS*), *leucoanthocyanidin reductase* (*LAR*), and *anthocyanidin reductase* (*ANR*), were downregulated in EL (Supplementary Figure S7). These metabolic changes in flavonoid and catechin biosynthesis also contributed to the high phenylalanine content (Figure 6), an initial precursor in these pathways and amino acids, and low catechin content in EL (Figure 3).

**Figure 8:**
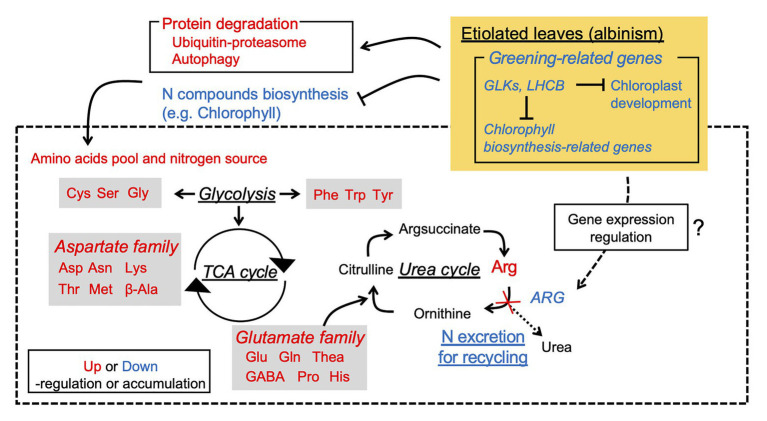
Hypothetical schematic model of albinism-induced accumulation of free amino acids in an etiolated tea cultivar.

The present study revealed the etiolation-induced metabolic changes leading to FAA accumulation from integrated omics analyses, from which a hypothetical schematic model of FAA accumulation is proposed (Figure 8). The high FAA contents in EL were largely a result of the increased FAA pool and N source arising from protein degradation, especially from degradation of chloroplasts and LHCs, and low N consumption rather than enhanced amino acid biosynthesis. Integrated analyses of the Chinese light-sensitive albino tea cultivar “Huangjinya” also indicated that a similar mechanism was in operation by comparison with shading treatment (Zhang et al., 2017). The present analyses, through comparisons with green leaf phenotypes induced by the BM or shading treatment, indicated that the etiolation-induced molecular response was independent of the light environment and was largely influenced by the reaction of the etiolation itself. In particular, the BM shared the same genetic background and light environment as the EL, thereby avoiding the confounding influence of genetic and environment differences, which is a limitation of previous studies of albino tea (Zhang et al., 2017, 2018). Additionally, the drastic downregulation of *ARG-1* might cause stagnation of the urea cycle and contribute to the upstream accumulation of FAAs. These findings will contribute to not only the breeding of high-quality tea but also the use of albino plants as agricultural crops.

## Data Availability Statement

The datasets presented in this study can be found in online repositories. The names of the repository/repositories and accession number(s) can be found in the article/Supplementary Material.

## Author Contributions

HY, YK, MO, AK, YT, and TI performed the experiment. TS and YN managed the tea plants. HY, YK, and YT analyzed the metabolome and transcriptome data. HY performed the data visualization. HY, AM, and TI wrote the manuscript. AM and TI designed the study and sourced funding. All authors read and approved the manuscript.

### Conflict of Interest

The authors declare that the research was conducted in the absence of any commercial or financial relationships that could be construed as a potential conflict of interest.
