# Supplementary material for: Integrated Metabolome and Transcriptome Analyses Reveal Etiolation-Induced Metabolic Changes Leading to High Amino Acid Contents in a Light-Sensitive Japanese Albino Tea Cultivar
Source: Front Plant Sci. 2021 Jan 18;11:611140. doi: 10.3389/fpls.2020.611140 (PMC7847902; doi:10.3389/fpls.2020.611140)
Supplement: Supplementary file 2 [file Data_Sheet_1.pdf]

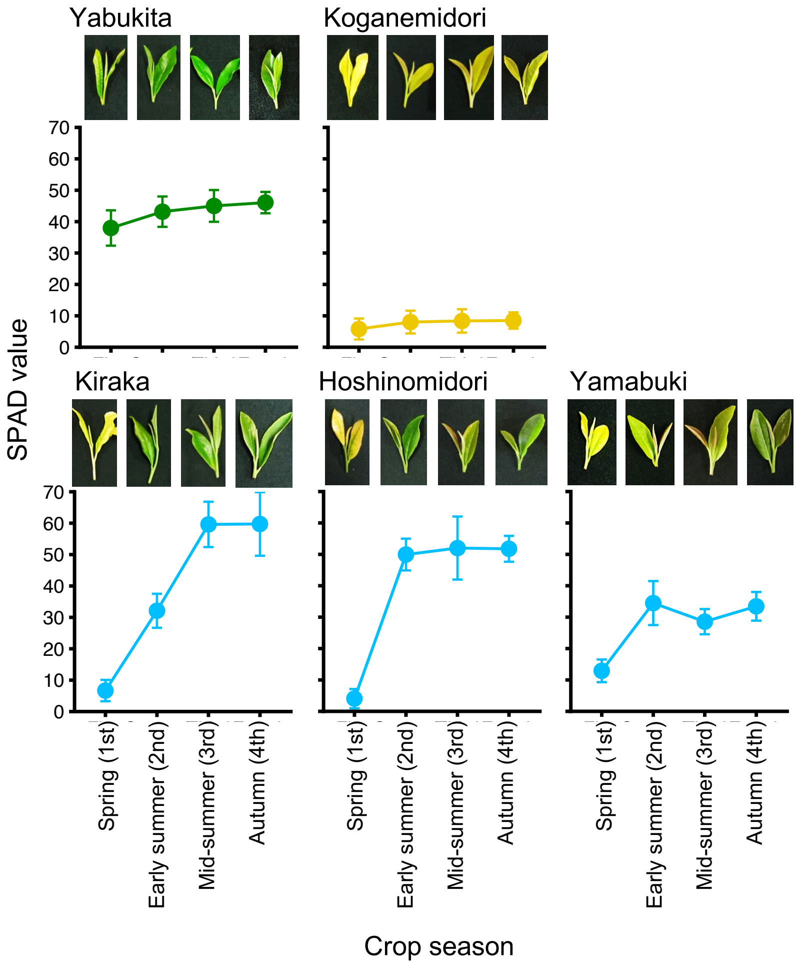

Supplementary Figure S1. Seasonal changes in green leaf phenotype in four albino tea cultivars in Japan.

The chlorophyll meter (SPAD) value was measured to evaluate the green color of new leaves of 'Koganemidori', 'Kiraka', 'Hoshinomidori', and 'Yamabuki' as representative albino tea cultivars. 'Yabukita', a leading Japanese green tea cultivar, was used as a reference. Data and error bars are the mean ± SD (*n* = 3).

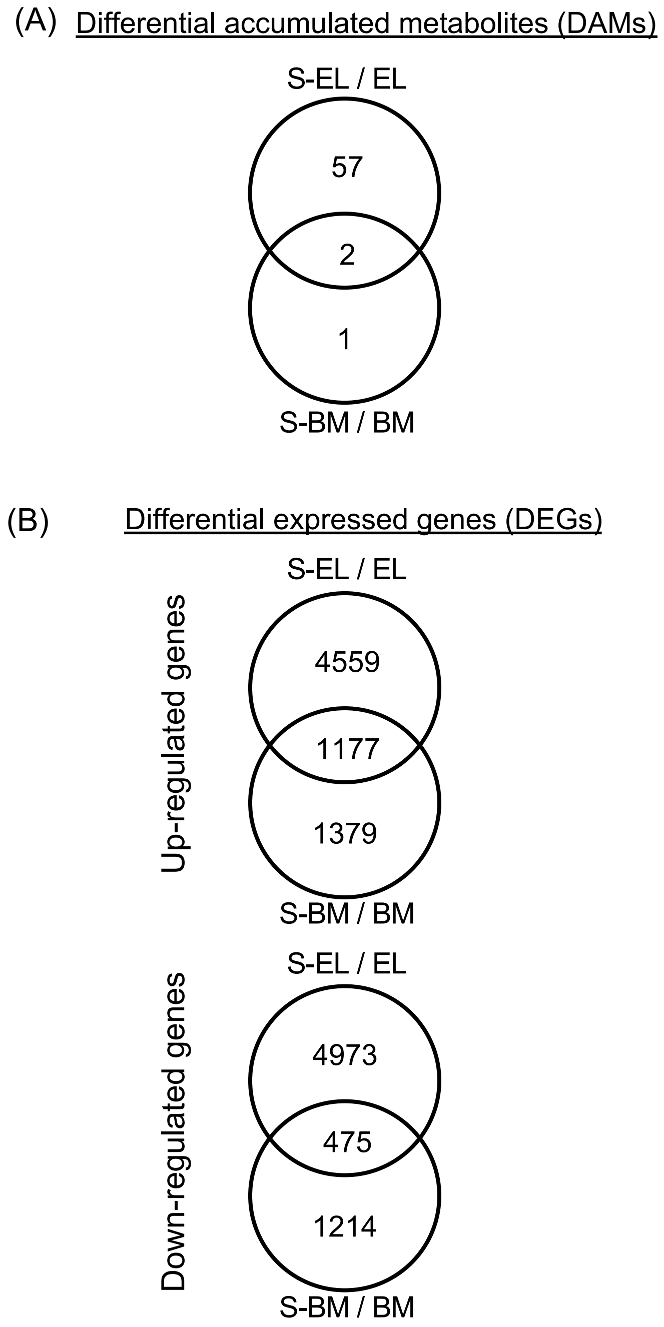

Supplementary Figure S2. Venn diagram analyses of differentially accumulated metabolites (DAMs; A) and differentially expressed genes (DEGs; B) between S-EL/EL and S-BM/BM.

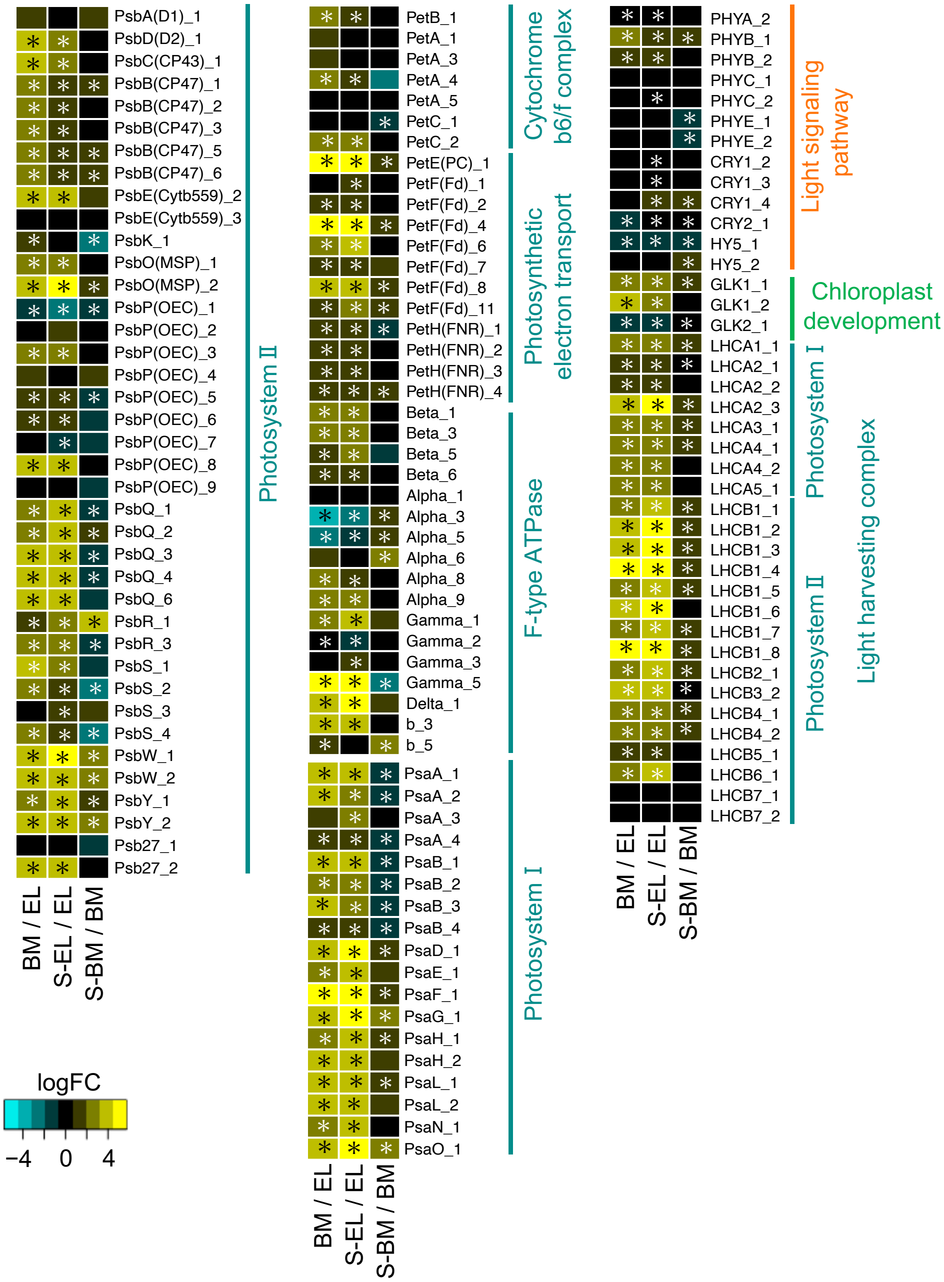

Supplementary Figure S3. Expression profiles of genes involved in photosynthesis, the light signaling pathway, and chloroplast development.

Heatmaps show log_2 fold change (log_2FC) of the expressed genes in the comparisons BM/EL (left), S-EL/EL (middle), and S-BM/BM (right). An asterisk in the boxes indicates a significant difference (FDR < 0.05).

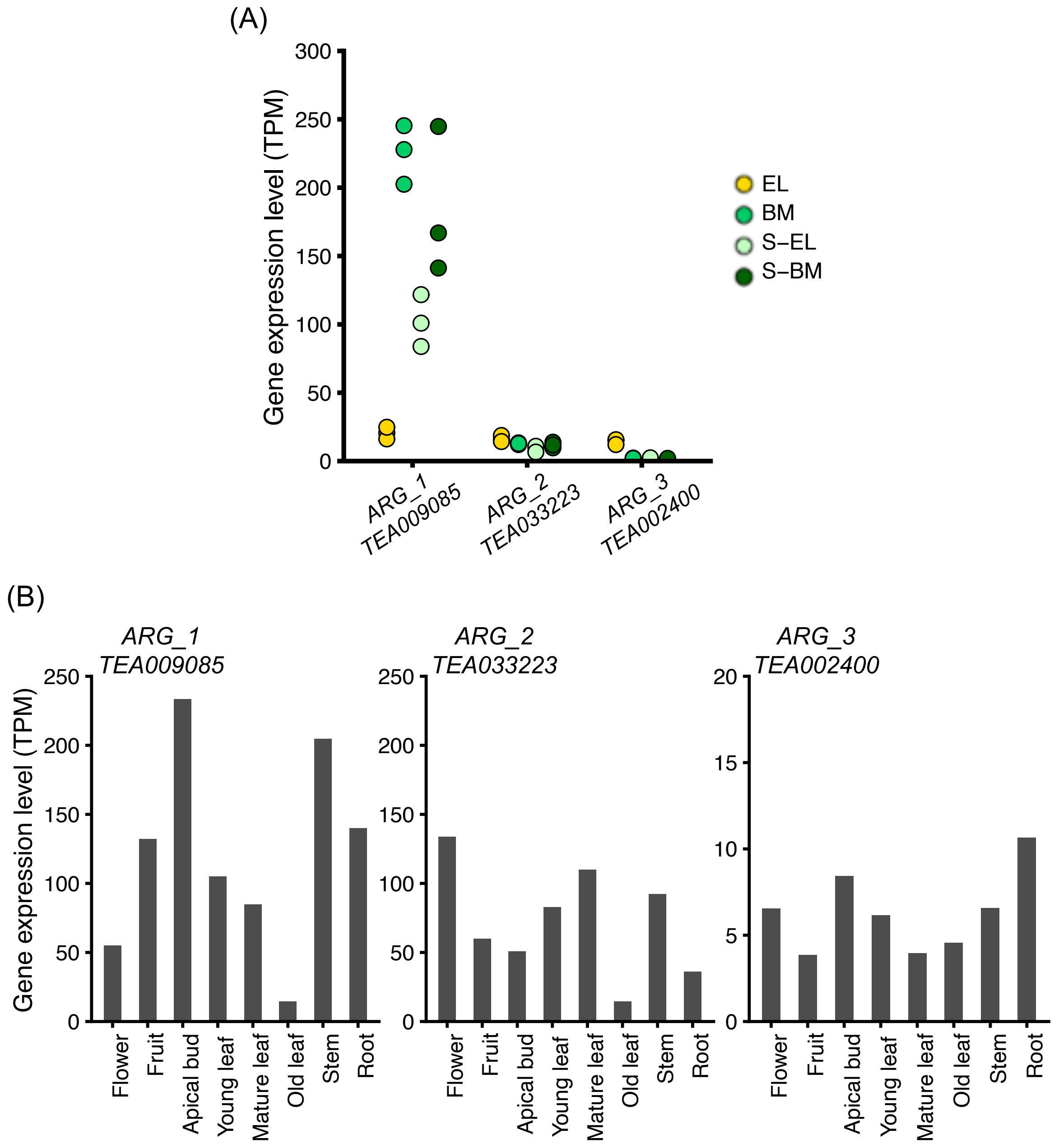

Supplementary Figure S4. Expression patterns of *CsARG* homologs.

Expression levels of *CsARG* homologs in AYL, GL-BM, GL-LL, and GL-BM-LL (A). Tissue expression profiles of *CsARG* homologs from the Tea Plant Information Archive (B).

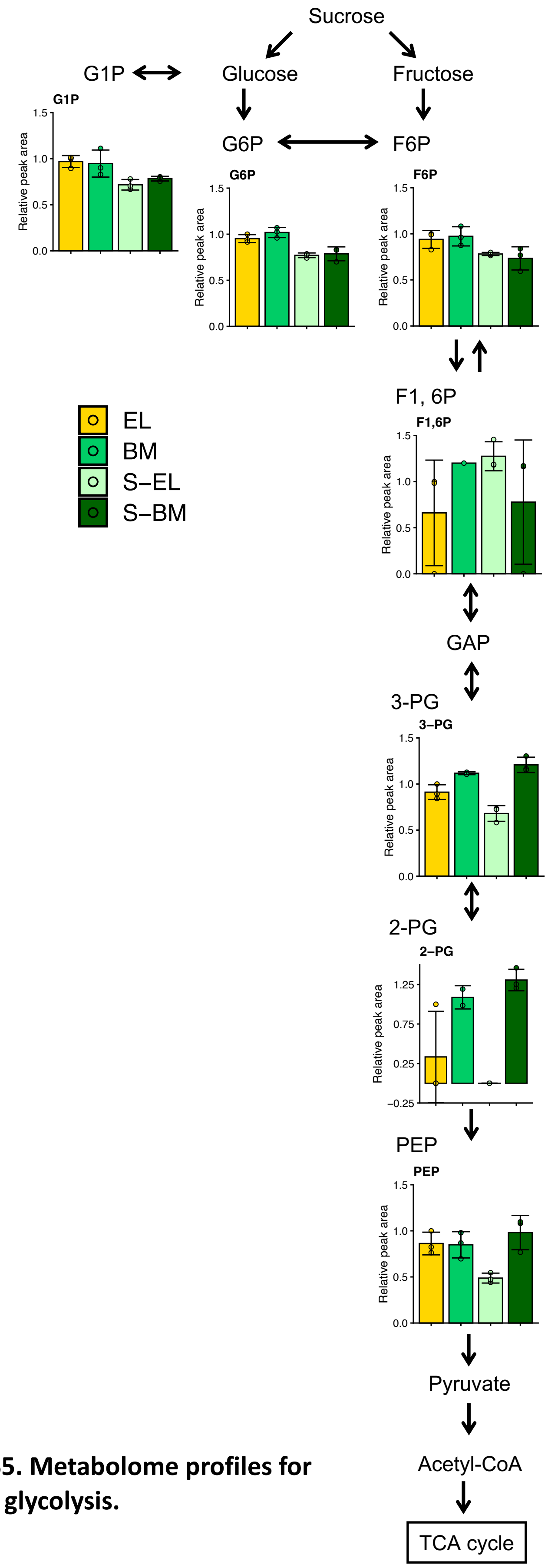

Supplementary Figure S5. Metabolome profiles for metabolites involved in glycolysis.

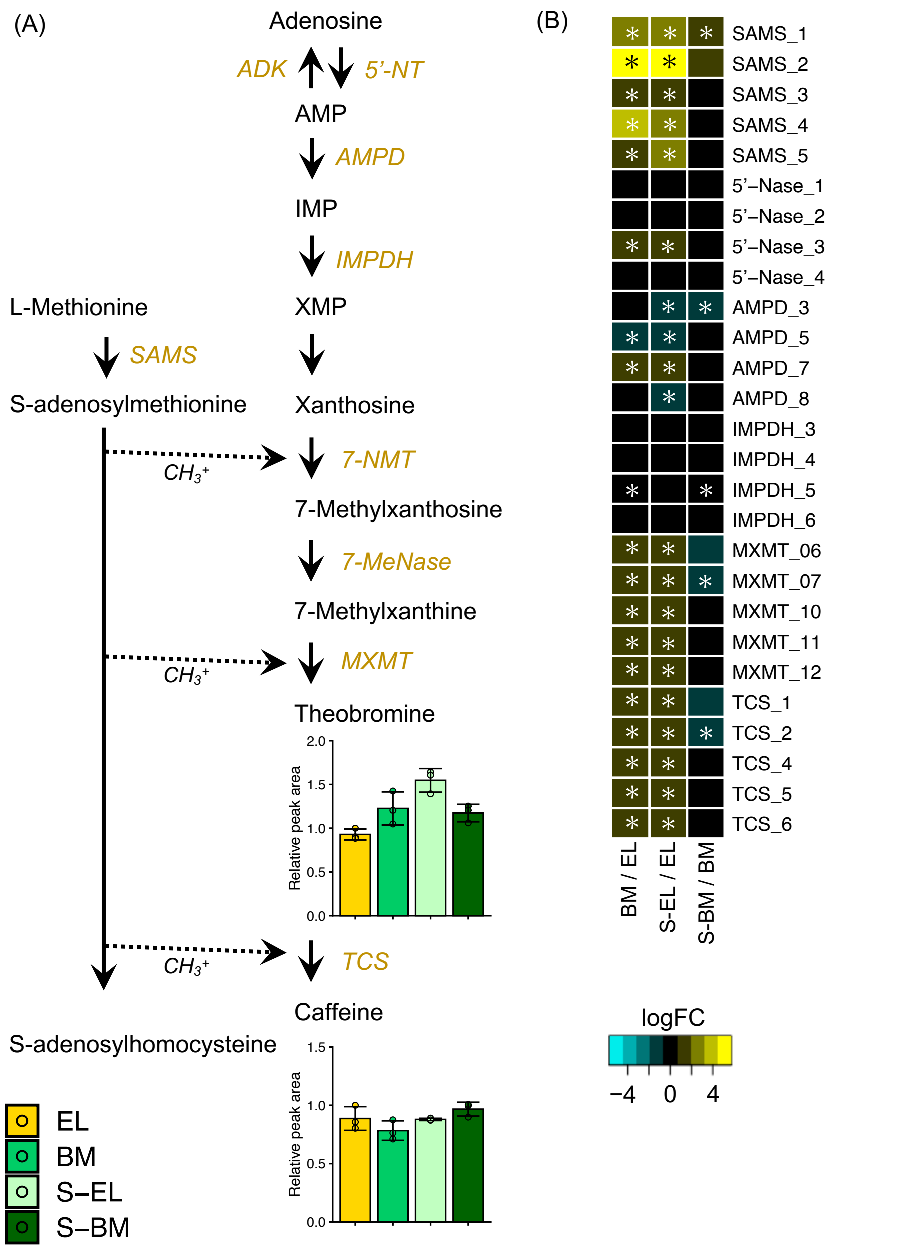

Supplementary Figure S6. Metabolome and expression profiles of genes involved in the caffeine biosynthesis pathway.

Heatmaps show $\log_2$ fold change ($\log_2$FC) of the expressed genes in the comparisons BM/EL (left), S-EL/EL (middle), and S-BM/BM (right). An asterisk in the boxes indicates a significant difference (FDR < 0.05).

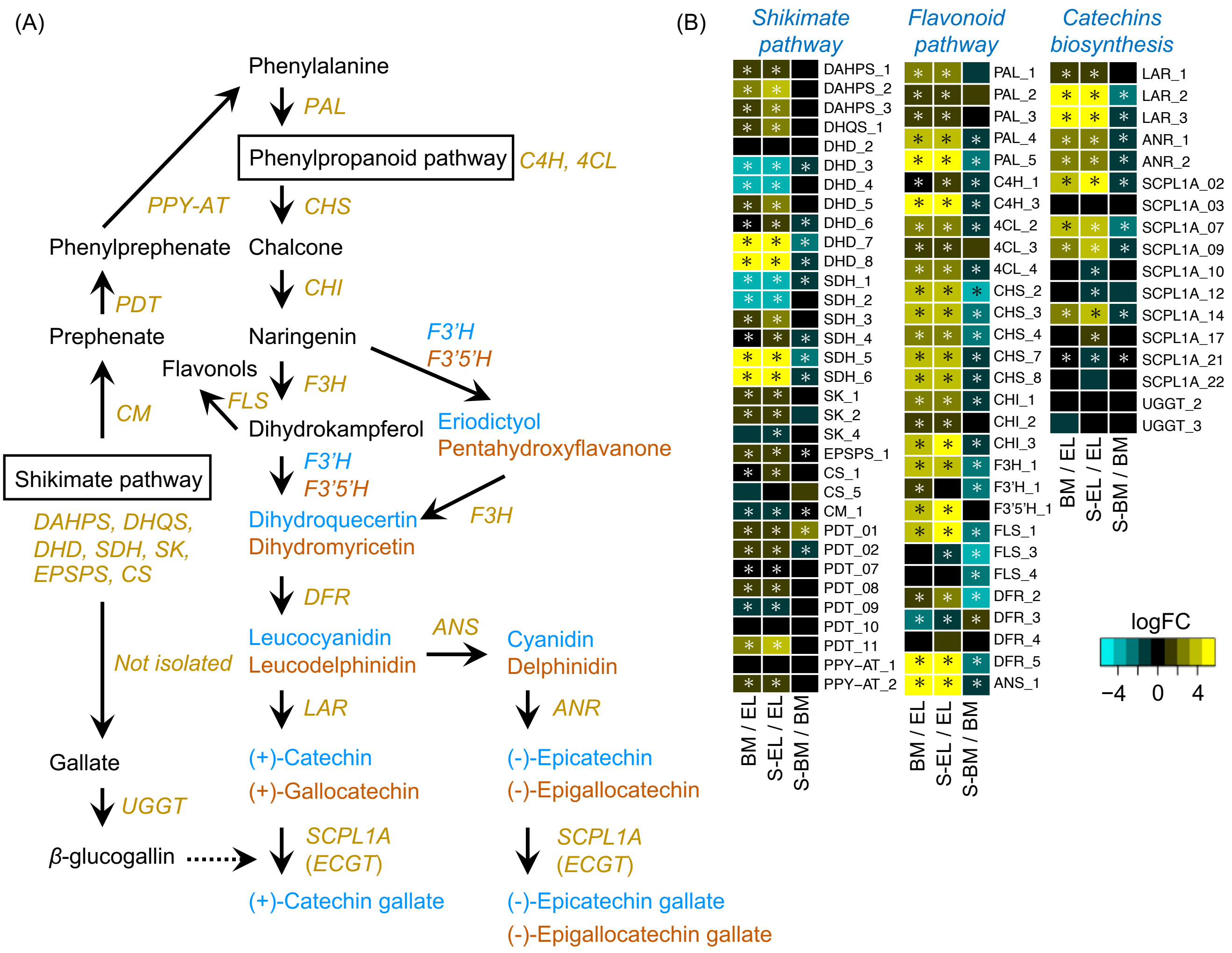

Supplementary Figure S7. Expression profiles of genes involved in the catechins biosynthesis pathway.

Heatmaps show $\log_2$ fold change ($\log_2$FC) of the expressed genes in the comparisons BM/EL (left), S-EL/EL (middle), and S-BM/BM (right). An asterisk in the boxes indicates a significant difference (FDR < 0.05).
